# Tetra­aqua­{1-[(1*H*-1,2,3-benzotriazol-1-yl)meth­yl]-1*H*-imidazole}­sulfato­manganese(II) dihydrate

**DOI:** 10.1107/S1600536811022197

**Published:** 2011-06-18

**Authors:** Ying Wang, Ying-Ying Sun

**Affiliations:** a Department of Geriatrics, The First Affiliated Hospital, Zhengzhou University, Zhengzhou 450000, People’s Republic of China; b Department of Pharmacy, The Third Affiliated Hospital of Henan University of Traditional Chinese Medicine, Zhengzhou 450008, People’s Republic of China

## Abstract

In the title complex, [Mn(SO_4_)(C_10_H_9_N_5_)(H_2_O)_4_]·2H_2_O, the Mn^2+^ cation is six-coordinated by one N atom from a 1-[(1*H*-1,2,3-benzotriazol-1-yl)meth­yl]-1*H*-imidazole ligand and five O atoms from one monodentate sulfate ligand and four water mol­ecules in a distorted octa­hedral geometry. In the crystal, adjacent mol­ecules are linked through O—H⋯O and O—H⋯N hydrogen bonds into a three-dimensional network.

## Related literature

For background to complexes based on flexible organic ligands, see: Ma *et al.* (2011 ▶); Meng *et al.* (2009 ▶); Sanchez *et al.* (2002 ▶).
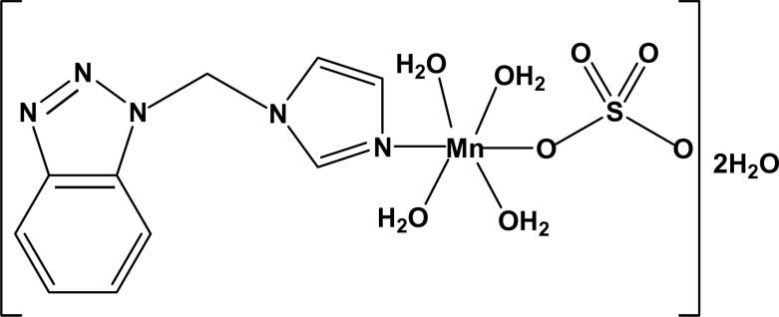


## Experimental

### 

#### Crystal data

[Mn(SO_4_)(C_10_H_9_N_5_)(H_2_O)_4_]·2H_2_O
*M* *_r_* = 458.32
Triclinic, 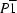

*a* = 7.5824 (15) Å
*b* = 8.5237 (17) Å
*c* = 15.972 (3) Å
α = 98.33 (3)°
β = 91.11 (3)°
γ = 115.21 (3)°
*V* = 920.3 (3) Å^3^
*Z* = 2
Mo *K*α radiation
μ = 0.89 mm^−1^
*T* = 293 K
0.20 × 0.18 × 0.15 mm

#### Data collection

Rigaku Saturn diffractometer
Absorption correction: multi-scan (*CrystalClear*; Rigaku/MSC, 2006 ▶) *T*_min_ = 0.842, *T*_max_ = 0.878
11432 measured reflections
4337 independent reflections
3890 reflections with *I* > 2σ(*I*)
*R*_int_ = 0.022

#### Refinement

*R*[*F* ^2^ > 2σ(*F* ^2^)] = 0.038
*wR*(*F* ^2^) = 0.087
*S* = 1.03
4337 reflections
244 parameters
H-atom parameters constrained
Δρ_max_ = 0.88 e Å^−3^
Δρ_min_ = −0.42 e Å^−3^

### 

Data collection: *CrystalClear* (Rigaku/MSC, 2006 ▶); cell refinement: *CrystalClear*; data reduction: *CrystalClear*; program(s) used to solve structure: *SHELXS97* (Sheldrick, 2008 ▶); program(s) used to refine structure: *SHELXL97* (Sheldrick, 2008 ▶); molecular graphics: *XP* in *SHELXTL* (Sheldrick, 2008 ▶); software used to prepare material for publication: *SHELXTL*.

## Supplementary Material

Crystal structure: contains datablock(s) global, I. DOI: 10.1107/S1600536811022197/wm2495sup1.cif

Structure factors: contains datablock(s) I. DOI: 10.1107/S1600536811022197/wm2495Isup2.hkl

Additional supplementary materials:  crystallographic information; 3D view; checkCIF report

## Figures and Tables

**Table 1:** Selected bond lengths (Å)

Mn1—O5	2.1543 (16)
Mn1—O8	2.1854 (15)
Mn1—O7	2.1860 (16)
Mn1—N1	2.2043 (17)
Mn1—O6	2.2142 (17)
Mn1—O1	2.2269 (16)

**Table 2:** Hydrogen-bond geometry (Å, °)

*D*—H⋯*A*	*D*—H	H⋯*A*	*D*⋯*A*	*D*—H⋯*A*
O5—H1*W*⋯O10	0.85	1.79	2.634 (2)	173
O5—H2*W*⋯O3	0.85	1.92	2.729 (3)	159
O9—H9*W*⋯O4	0.85	2.02	2.843 (3)	163
O6—H3*W*⋯O9^i^	0.85	1.98	2.824 (2)	170
O8—H8*W*⋯O1^i^	0.85	2.04	2.885 (2)	176
O7—H6*W*⋯O4^i^	0.85	2.03	2.855 (3)	163
O6—H4*W*⋯O4^ii^	0.85	1.96	2.805 (2)	173
O7—H5*W*⋯O9^ii^	0.85	1.99	2.813 (2)	162
O8—H7*W*⋯O2^iii^	0.85	1.87	2.712 (2)	172
O10—H11*W*⋯O2^iii^	0.85	2.08	2.842 (3)	150
O10—H12*W*⋯N5^iv^	0.85	1.99	2.840 (3)	173
O9—H10*W*⋯O1^v^	0.85	2.24	3.083 (2)	173

Symmetry codes: (i) 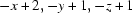; (ii) 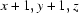; (iii) 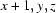; (iv) 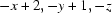; (v) 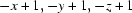.

**Fig. 1.**
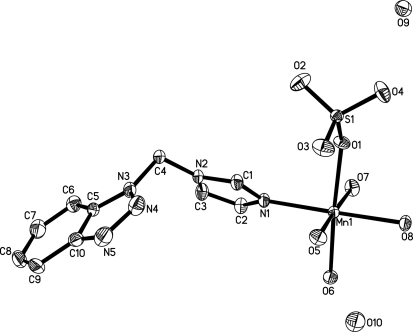
View of the title complex, showing the labelling of the 30% probability ellipsoids. H atoms are omitted for clarity.

**Fig. 2.**
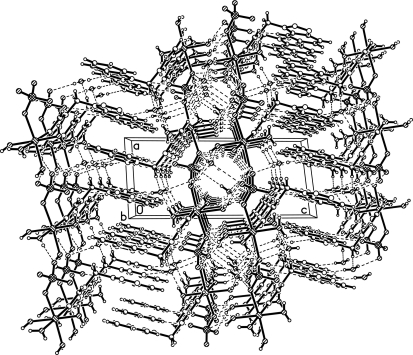
Packing plot of the title complex, showing the hydrogen bonding (dashed lines)

## supplementary crystallographic information

### Comment

A large number of metal-organic frameworks based on flexible organic ligands have been reported since they are good linkers and can influence the structural diversification of the products, including the formation of supramolecular isomers (Ma *et al.*, 2011; Meng *et al.*, 2009; Sanchez *et al.*, 2002). In order to further explore complexes with novel structures, in this work, through the reaction of 1-[1*H*-1,2,3-benzotriazol-1-yl)methyl]-1*H*-1,3-imidazole (bmi) with manganese sulfate at room temperature, we obtained the title complex, [Mn(SO_4_)(C_10_H_9_N_5_)(H_2_O)_4_](H_2_O)_2_, which is reported here.

As shown in Figure 1, the Mn(II) ion features a distorted octahedral coordination geometry and is surrounded by five oxygen atoms from four water molecules and one monodentate sulfate ligand as well as one nitrogen atom from the bmi ligand. Atoms O1, O5, O6, O7 form the equatorial plane, whereas O8 and N1 atoms are located in the apical positions. The bond angle of O(8)—Mn(1)—N(1) is 176.71 (6) °. Intramolecular O—H···O hydrogen bonds stabilize the molecular configuration and O—H···O, O—H···N hydrogen bonds between adjacent molecules consolidate the crystal packing (Fig. 2).

### Experimental

The ligand 1-[1*H*-1,2,3-benzotriazol-1-yl)methyl]-1*H*-1,3-imidazole (0.1 mmol) in methanol (4 ml) was added dropwise to an aqueous solution (2 ml) of manganese sulfate (0.1 mmol). The resulting solution was allowed to stand at room temperature. After four weeks colorless crystals with good quality were obtained from the filtrate and dried in air.

### Refinement

H atoms are positioned geometrically and refined as riding atoms, with C-H = 0.93 (aromatic) and 0.97 (CH_2_) Å and O-H = 0.85 Å, and with U_iso_(H) = 1.2 U_eq_(C,O).

### Crystal data

**Table:**

[Mn(SO_4_)(C_10_H_9_N_5_)(H_2_O)_4_]·2H_2_O	*Z* = 2
*M**_r_* = 458.32	*F*(000) = 474
Triclinic, *P*1	*D*_x_ = 1.654 Mg m^−^^3^
Hall symbol: -P 1	Mo *K*α radiation, λ = 0.71073 Å
*a* = 7.5824 (15) Å	Cell parameters from 2952 reflections
*b* = 8.5237 (17) Å	θ = 2.6–27.9°
*c* = 15.972 (3) Å	µ = 0.89 mm^−^^1^
α = 98.33 (3)°	*T* = 293 K
β = 91.11 (3)°	Prism, colourless
γ = 115.21 (3)°	0.20 × 0.18 × 0.15 mm
*V* = 920.3 (3) Å^3^	

### Data collection

**Table:**

Rigaku Saturn diffractometer	4337 independent reflections
Radiation source: fine-focus sealed tube	3890 reflections with *I* > 2σ(*I*)
graphite	*R*_int_ = 0.022
Detector resolution: 28.5714 pixels mm^-1^	θ_max_ = 27.9°, θ_min_ = 2.6°
ω scans	*h* = −9→9
Absorption correction: multi-scan (*CrystalClear*; Rigaku/MSC, 2006)	*k* = −9→11
*T*_min_ = 0.842, *T*_max_ = 0.878	*l* = −20→20
11432 measured reflections	

### Refinement

**Table:**

Refinement on *F*^2^	Primary atom site location: structure-invariant direct methods
Least-squares matrix: full	Secondary atom site location: difference Fourier map
*R*[*F*^2^ > 2σ(*F*^2^)] = 0.038	Hydrogen site location: inferred from neighbouring sites
*wR*(*F*^2^) = 0.087	H-atom parameters constrained
*S* = 1.03	*w* = 1/[σ^2^(*F*_o_^2^) + (0.037*P*)^2^ + 0.6298*P*] where *P* = (*F*_o_^2^ + 2*F*_c_^2^)/3
4337 reflections	(Δ/σ)_max_ < 0.001
244 parameters	Δρ_max_ = 0.88 e Å^−^^3^
0 restraints	Δρ_min_ = −0.42 e Å^−^^3^

### Special details

**Table:**

Geometry. All e.s.d.'s (except the e.s.d. in the dihedral angle between two l.s. planes) are estimated using the full covariance matrix. The cell e.s.d.'s are taken into account individually in the estimation of e.s.d.'s in distances, angles and torsion angles; correlations between e.s.d.'s in cell parameters are only used when they are defined by crystal symmetry. An approximate (isotropic) treatment of cell e.s.d.'s is used for estimating e.s.d.'s involving l.s. planes.
Refinement. Refinement of *F*^2^ against ALL reflections. The weighted *R*-factor *wR* and goodness of fit *S* are based on *F*^2^, conventional *R*-factors *R* are based on *F*, with *F* set to zero for negative *F*^2^. The threshold expression of *F*^2^ > σ(*F*^2^) is used only for calculating *R*-factors(gt) *etc*. and is not relevant to the choice of reflections for refinement. *R*-factors based on *F*^2^ are statistically about twice as large as those based on *F*, and *R*- factors based on ALL data will be even larger.

### Fractional atomic coordinates and isotropic or equivalent isotropic displacement parameters (Å^2^)

**Table:**

	*x*	*y*	*z*	*U*_iso_*/*U*_eq_	
Mn1	1.09479 (4)	0.68072 (4)	0.368939 (17)	0.02329 (9)	
N1	0.9825 (2)	0.8445 (2)	0.31175 (11)	0.0291 (4)	
N2	0.7818 (2)	0.9044 (2)	0.23568 (10)	0.0286 (4)	
N3	0.6756 (3)	0.8884 (2)	0.09051 (11)	0.0335 (4)	
N4	0.6786 (3)	0.7462 (3)	0.04108 (13)	0.0463 (5)	
N5	0.7294 (3)	0.7876 (3)	−0.03268 (13)	0.0502 (5)	
O1	0.7941 (2)	0.51495 (19)	0.40066 (9)	0.0329 (3)	
O2	0.4595 (3)	0.4090 (3)	0.34648 (13)	0.0563 (5)	
O3	0.6674 (3)	0.3117 (2)	0.26847 (10)	0.0474 (4)	
O4	0.5682 (3)	0.2154 (2)	0.40145 (11)	0.0477 (4)	
O5	1.0451 (2)	0.5238 (2)	0.24469 (9)	0.0369 (3)	
H1W	1.1290	0.4924	0.2231	0.044*	
H2W	0.9310	0.4400	0.2415	0.044*	
O6	1.4025 (2)	0.84945 (19)	0.34976 (10)	0.0354 (3)	
H3W	1.4870	0.8318	0.3776	0.042*	
H4W	1.4499	0.9610	0.3610	0.042*	
O7	1.1386 (2)	0.8208 (2)	0.49917 (9)	0.0391 (4)	
H5W	1.1708	0.9306	0.5100	0.047*	
H6W	1.2073	0.8024	0.5358	0.047*	
O8	1.1938 (2)	0.5052 (2)	0.42073 (10)	0.0380 (4)	
H7W	1.2767	0.4702	0.4012	0.046*	
H8W	1.1915	0.4943	0.4728	0.046*	
O9	0.3306 (2)	0.1900 (2)	0.53910 (10)	0.0399 (4)	
H9W	0.4033	0.2189	0.4988	0.048*	
H10W	0.3046	0.2747	0.5596	0.048*	
O10	1.3276 (3)	0.4486 (2)	0.18654 (11)	0.0510 (4)	
H11W	1.3663	0.4031	0.2225	0.061*	
H12W	1.3172	0.3753	0.1423	0.061*	
S1	0.62136 (7)	0.36114 (6)	0.35346 (3)	0.02383 (11)	
C1	0.8275 (3)	0.7791 (3)	0.25673 (14)	0.0323 (4)	
H1A	0.7579	0.6599	0.2350	0.039*	
C2	1.0396 (3)	1.0233 (3)	0.32650 (14)	0.0349 (5)	
H2A	1.1463	1.1055	0.3629	0.042*	
C3	0.9168 (4)	1.0606 (3)	0.27995 (15)	0.0392 (5)	
H3A	0.9230	1.1714	0.2783	0.047*	
C4	0.6192 (3)	0.8766 (3)	0.17646 (13)	0.0364 (5)	
H4A	0.5134	0.7615	0.1773	0.044*	
H4B	0.5714	0.9640	0.1945	0.044*	
C5	0.7249 (3)	1.0253 (3)	0.04678 (12)	0.0309 (4)	
C6	0.7348 (3)	1.1929 (3)	0.06646 (15)	0.0409 (5)	
H6A	0.7078	1.2356	0.1192	0.049*	
C7	0.7875 (4)	1.2921 (4)	0.00210 (19)	0.0544 (7)	
H7A	0.7978	1.4061	0.0120	0.065*	
C8	0.8259 (4)	1.2264 (5)	−0.07757 (19)	0.0605 (8)	
H8A	0.8619	1.2985	−0.1187	0.073*	
C9	0.8125 (4)	1.0620 (5)	−0.09655 (16)	0.0546 (7)	
H9A	0.8369	1.0195	−0.1499	0.065*	
C10	0.7603 (3)	0.9581 (3)	−0.03262 (13)	0.0391 (5)	

### Atomic displacement parameters (Å^2^)

**Table:**

	*U*^11^	*U*^22^	*U*^33^	*U*^12^	*U*^13^	*U*^23^
Mn1	0.02459 (15)	0.02428 (16)	0.02242 (15)	0.01142 (12)	0.00181 (11)	0.00560 (11)
N1	0.0309 (9)	0.0280 (9)	0.0306 (9)	0.0139 (7)	−0.0007 (7)	0.0086 (7)
N2	0.0300 (9)	0.0348 (9)	0.0258 (8)	0.0169 (7)	0.0011 (7)	0.0107 (7)
N3	0.0370 (10)	0.0413 (10)	0.0267 (9)	0.0203 (8)	0.0001 (7)	0.0085 (8)
N4	0.0532 (13)	0.0501 (12)	0.0432 (11)	0.0313 (11)	−0.0020 (10)	0.0033 (10)
N5	0.0543 (13)	0.0665 (15)	0.0378 (11)	0.0376 (12)	−0.0010 (10)	−0.0032 (10)
O1	0.0280 (7)	0.0301 (8)	0.0295 (7)	0.0027 (6)	0.0038 (6)	0.0030 (6)
O2	0.0394 (10)	0.0640 (12)	0.0746 (13)	0.0333 (9)	0.0045 (9)	0.0049 (10)
O3	0.0467 (10)	0.0523 (10)	0.0286 (8)	0.0102 (8)	0.0061 (7)	−0.0020 (7)
O4	0.0593 (11)	0.0292 (8)	0.0443 (9)	0.0072 (8)	−0.0041 (8)	0.0141 (7)
O5	0.0410 (9)	0.0341 (8)	0.0321 (8)	0.0144 (7)	0.0066 (6)	0.0007 (6)
O6	0.0276 (7)	0.0313 (8)	0.0443 (9)	0.0088 (6)	0.0024 (6)	0.0096 (7)
O7	0.0515 (10)	0.0356 (8)	0.0262 (7)	0.0178 (7)	−0.0043 (7)	−0.0021 (6)
O8	0.0497 (9)	0.0482 (9)	0.0346 (8)	0.0354 (8)	0.0111 (7)	0.0171 (7)
O9	0.0427 (9)	0.0342 (8)	0.0448 (9)	0.0181 (7)	0.0105 (7)	0.0071 (7)
O10	0.0652 (12)	0.0600 (11)	0.0368 (9)	0.0376 (10)	0.0053 (8)	0.0019 (8)
S1	0.0226 (2)	0.0225 (2)	0.0259 (2)	0.00928 (18)	0.00274 (17)	0.00440 (18)
C1	0.0345 (11)	0.0261 (10)	0.0348 (11)	0.0112 (8)	−0.0036 (9)	0.0076 (8)
C2	0.0382 (11)	0.0275 (10)	0.0353 (11)	0.0121 (9)	−0.0060 (9)	0.0024 (9)
C3	0.0492 (14)	0.0281 (11)	0.0437 (13)	0.0201 (10)	−0.0024 (10)	0.0071 (9)
C4	0.0312 (11)	0.0560 (14)	0.0296 (10)	0.0226 (10)	0.0040 (8)	0.0180 (10)
C5	0.0262 (10)	0.0422 (12)	0.0241 (9)	0.0139 (9)	−0.0011 (7)	0.0083 (8)
C6	0.0378 (12)	0.0407 (13)	0.0371 (12)	0.0119 (10)	−0.0067 (10)	0.0021 (10)
C7	0.0431 (14)	0.0435 (14)	0.0670 (18)	0.0068 (11)	−0.0070 (13)	0.0209 (13)
C8	0.0390 (14)	0.085 (2)	0.0531 (16)	0.0126 (14)	0.0050 (12)	0.0427 (16)
C9	0.0408 (14)	0.094 (2)	0.0303 (12)	0.0269 (14)	0.0104 (10)	0.0222 (13)
C10	0.0309 (11)	0.0607 (15)	0.0266 (10)	0.0216 (10)	0.0025 (8)	0.0047 (10)

### Geometric parameters (Å, °)

**Table:**

Mn1—O5	2.1543 (16)	O7—H5W	0.8500
Mn1—O8	2.1854 (15)	O7—H6W	0.8499
Mn1—O7	2.1860 (16)	O8—H7W	0.8500
Mn1—N1	2.2043 (17)	O8—H8W	0.8500
Mn1—O6	2.2142 (17)	O9—H9W	0.8500
Mn1—O1	2.2269 (16)	O9—H10W	0.8501
N1—C1	1.313 (3)	O10—H11W	0.8499
N1—C2	1.377 (3)	O10—H12W	0.8500
N2—C1	1.338 (3)	C1—H1A	0.9300
N2—C3	1.363 (3)	C2—C3	1.347 (3)
N2—C4	1.453 (3)	C2—H2A	0.9300
N3—N4	1.357 (3)	C3—H3A	0.9300
N3—C5	1.365 (3)	C4—H4A	0.9700
N3—C4	1.450 (3)	C4—H4B	0.9700
N4—N5	1.297 (3)	C5—C6	1.387 (3)
N5—C10	1.369 (3)	C5—C10	1.394 (3)
O1—S1	1.4885 (16)	C6—C7	1.382 (4)
O2—S1	1.4560 (17)	C6—H6A	0.9300
O3—S1	1.4561 (16)	C7—C8	1.402 (4)
O4—S1	1.4677 (17)	C7—H7A	0.9300
O5—H1W	0.8501	C8—C9	1.349 (4)
O5—H2W	0.8500	C8—H8A	0.9300
O6—H3W	0.8500	C9—C10	1.401 (3)
O6—H4W	0.8500	C9—H9A	0.9300
			
O5—Mn1—O8	89.65 (6)	H11W—O10—H12W	98.3
O5—Mn1—O7	175.48 (6)	O2—S1—O3	108.94 (12)
O8—Mn1—O7	86.58 (6)	O2—S1—O4	109.81 (12)
O5—Mn1—N1	87.70 (7)	O3—S1—O4	110.79 (11)
O8—Mn1—N1	176.71 (6)	O2—S1—O1	108.90 (11)
O7—Mn1—N1	95.98 (7)	O3—S1—O1	110.12 (10)
O5—Mn1—O6	92.32 (7)	O4—S1—O1	108.25 (10)
O8—Mn1—O6	88.81 (6)	N1—C1—N2	111.90 (19)
O7—Mn1—O6	90.10 (7)	N1—C1—H1A	124.1
N1—Mn1—O6	93.25 (7)	N2—C1—H1A	124.1
O5—Mn1—O1	92.08 (7)	C3—C2—N1	109.40 (19)
O8—Mn1—O1	88.47 (6)	C3—C2—H2A	125.3
O7—Mn1—O1	85.33 (7)	N1—C2—H2A	125.3
N1—Mn1—O1	89.68 (7)	C2—C3—N2	106.71 (19)
O6—Mn1—O1	174.81 (6)	C2—C3—H3A	126.6
C1—N1—C2	105.20 (17)	N2—C3—H3A	126.6
C1—N1—Mn1	123.33 (14)	N3—C4—N2	111.86 (17)
C2—N1—Mn1	131.33 (14)	N3—C4—H4A	109.2
C1—N2—C3	106.80 (17)	N2—C4—H4A	109.2
C1—N2—C4	125.85 (19)	N3—C4—H4B	109.2
C3—N2—C4	127.36 (19)	N2—C4—H4B	109.2
N4—N3—C5	110.75 (18)	H4A—C4—H4B	107.9
N4—N3—C4	119.50 (19)	N3—C5—C6	133.4 (2)
C5—N3—C4	129.72 (19)	N3—C5—C10	103.5 (2)
N5—N4—N3	108.1 (2)	C6—C5—C10	123.0 (2)
N4—N5—C10	109.1 (2)	C7—C6—C5	115.2 (2)
S1—O1—Mn1	134.35 (9)	C7—C6—H6A	122.4
Mn1—O5—H1W	123.1	C5—C6—H6A	122.4
Mn1—O5—H2W	106.7	C6—C7—C8	122.1 (3)
H1W—O5—H2W	113.6	C6—C7—H7A	119.0
Mn1—O6—H3W	114.6	C8—C7—H7A	119.0
Mn1—O6—H4W	121.6	C9—C8—C7	122.2 (2)
H3W—O6—H4W	100.5	C9—C8—H8A	118.9
Mn1—O7—H5W	121.8	C7—C8—H8A	118.9
Mn1—O7—H6W	118.3	C8—C9—C10	117.3 (2)
H5W—O7—H6W	105.2	C8—C9—H9A	121.4
Mn1—O8—H7W	127.1	C10—C9—H9A	121.4
Mn1—O8—H8W	123.0	N5—C10—C5	108.5 (2)
H7W—O8—H8W	105.9	N5—C10—C9	131.3 (2)
H9W—O9—H10W	109.6	C5—C10—C9	120.2 (2)
			
O5—Mn1—N1—C1	42.94 (17)	N1—C2—C3—N2	0.0 (3)
O7—Mn1—N1—C1	−134.43 (17)	C1—N2—C3—C2	−0.1 (3)
O6—Mn1—N1—C1	135.13 (17)	C4—N2—C3—C2	−179.8 (2)
O1—Mn1—N1—C1	−49.16 (17)	N4—N3—C4—N2	82.7 (3)
O5—Mn1—N1—C2	−142.02 (19)	C5—N3—C4—N2	−99.3 (3)
O7—Mn1—N1—C2	40.6 (2)	C1—N2—C4—N3	−91.1 (3)
O6—Mn1—N1—C2	−49.82 (19)	C3—N2—C4—N3	88.5 (3)
O1—Mn1—N1—C2	125.89 (19)	N4—N3—C5—C6	176.6 (2)
C5—N3—N4—N5	0.4 (3)	C4—N3—C5—C6	−1.5 (4)
C4—N3—N4—N5	178.76 (19)	N4—N3—C5—C10	−0.6 (2)
N3—N4—N5—C10	0.0 (3)	C4—N3—C5—C10	−178.7 (2)
O5—Mn1—O1—S1	−2.77 (13)	N3—C5—C6—C7	−178.3 (2)
O8—Mn1—O1—S1	−92.36 (13)	C10—C5—C6—C7	−1.6 (3)
O7—Mn1—O1—S1	−179.05 (13)	C5—C6—C7—C8	0.7 (4)
N1—Mn1—O1—S1	84.92 (13)	C6—C7—C8—C9	0.5 (4)
Mn1—O1—S1—O2	−118.40 (14)	C7—C8—C9—C10	−0.8 (4)
Mn1—O1—S1—O3	0.99 (17)	N4—N5—C10—C5	−0.3 (3)
Mn1—O1—S1—O4	122.26 (14)	N4—N5—C10—C9	−178.4 (2)
C2—N1—C1—N2	−0.2 (2)	N3—C5—C10—N5	0.5 (2)
Mn1—N1—C1—N2	175.94 (13)	C6—C5—C10—N5	−177.0 (2)
C3—N2—C1—N1	0.2 (2)	N3—C5—C10—C9	178.9 (2)
C4—N2—C1—N1	179.91 (18)	C6—C5—C10—C9	1.4 (3)
C1—N1—C2—C3	0.1 (3)	C8—C9—C10—N5	177.8 (3)
Mn1—N1—C2—C3	−175.60 (16)	C8—C9—C10—C5	−0.1 (4)

### Hydrogen-bond geometry (Å, °)

**Table:**

*D*—H···*A*	*D*—H	H···*A*	*D*···*A*	*D*—H···*A*
O5—H1W···O10	0.85	1.79	2.634 (2)	173.
O5—H2W···O3	0.85	1.92	2.729 (3)	159.
O9—H9W···O4	0.85	2.02	2.843 (3)	163.
O6—H3W···O9^i^	0.85	1.98	2.824 (2)	170.
O8—H8W···O1^i^	0.85	2.04	2.885 (2)	176.
O7—H6W···O4^i^	0.85	2.03	2.855 (3)	163.
O6—H4W···O4^ii^	0.85	1.96	2.805 (2)	173.
O7—H5W···O9^ii^	0.85	1.99	2.813 (2)	162.
O8—H7W···O2^iii^	0.85	1.87	2.712 (2)	172.
O10—H11W···O2^iii^	0.85	2.08	2.842 (3)	150.
O10—H12W···N5^iv^	0.85	1.99	2.840 (3)	173.
O9—H10W···O1^v^	0.85	2.24	3.083 (2)	173.

Symmetry codes: (i) −*x*+2, −*y*+1, −*z*+1; (ii) *x*+1, *y*+1, *z*; (iii) *x*+1, *y*, *z*; (iv) −*x*+2, −*y*+1, −*z*; (v) −*x*+1, −*y*+1, −*z*+1.
